# Inactivation of NMD increases viability of *sup45 *nonsense mutants in *Saccharomyces cerevisiae*

**DOI:** 10.1186/1471-2199-8-71

**Published:** 2007-08-16

**Authors:** Svetlana Chabelskaya, Valentina Gryzina, Svetlana Moskalenko, Catherine Le Goff, Galina Zhouravleva

**Affiliations:** 1 Department of Genetics and Breeding, St Petersburg State University, Universitetskaya emb. 7/9, 199034, St Petersburg, Russia; 2 CNRS UMR 6061 Génétique et Développement, Université de Rennes 1, IFR 140, Faculté de Médecine, 2 av. Pr. Léon Bernard, CS 34317, 35043 Rennes Cedex, France

## Abstract

**Background:**

The nonsense-mediated mRNA decay (NMD) pathway promotes the rapid degradation of mRNAs containing premature termination codons (PTCs). In yeast *Saccharomyces cerevisiae*, the activity of the NMD pathway depends on the recognition of the PTC by the translational machinery. Translation termination factors eRF1 (Sup45) and eRF3 (Sup35) participate not only in the last step of protein synthesis but also in mRNA degradation and translation initiation *via *interaction with such proteins as Pab1, Upf1, Upf2 and Upf3.

**Results:**

In this work we have used previously isolated *sup45 *mutants of *S. cerevisiae *to characterize degradation of aberrant mRNA in conditions when translation termination is impaired. We have sequenced *his7-1*, *lys9-A21 *and *trp1-289 *alleles which are frequently used for analysis of nonsense suppression. We have established that *sup45 *nonsense and missense mutations lead to accumulation of *his7-1 *mRNA and *CYH2 *pre-mRNA. Remarkably, deletion of the *UPF1 *gene suppresses some *sup45 *phenotypes. In particular, *sup45-n upf1Δ *double mutants were less temperature sensitive, and more resistant to paromomycin than *sup45 *single mutants. In addition, deletion of either *UPF2 *or *UPF3 *restored viability of *sup45-n *double mutants.

**Conclusion:**

This is the first demonstration that *sup45 *mutations do not only change translation fidelity but also acts by causing a change in mRNA stability.

## Background

Two translation termination factors, eRF1 and eRF3, participate in termination of protein synthesis in eukaryotes (reviewed in [1]). In *S. cerevisiae *they are encoded by *SUP45 *and *SUP35*, respectively (reviewed in [2]). In eukaryotes, a single factor, eRF1 (Sup45 in yeast), decodes all three stop codons, while eRF3 (Sup35 in yeast) stimulates termination through a GTP-dependent mechanism by forming a complex with eRF1.

Eukaryotic cells possess a mechanism known as nonsense-mediated mRNA decay (NMD) to recognize and degrade mRNA molecules that contain premature termination codon (PTC) (reviewed in [3-5]). The NMD process is mediated by the *trans-*acting factors Upf1, Upf2 and Upf3 [6-11], all of which directly interact with eRF3; while only Upf1 interacts with eRF1 [12,13]. Using *in vitro *competition experiments, it has been demonstrated that Upf2, Upf3 and eRF1 actually compete with each other for binding to eRF3 [13]. Deletion of any one of the three *UPF *genes selectively stabilizes mRNAs that are degraded by the NMD pathway without affecting other mRNAs [6,7,9-11]. Genetic studies have shown that Upf1, Upf2, and Upf3 act as obligate partners in the NMD pathway; this means that NMD only occurs when all components are present (reviewed in [14,15]). Mutations or deletions of *UPF *genes lead to an increased frequency of nonsense suppression at termination codons in a variety of yeast genes (reviewed in [15]). A mutation in the GTP-binding motifs of eRF3 impairs the eRF1-binding ability and not only causes a defect in translation termination but also slows normal and nonsense-mediated mRNA decay, suggesting that GTP/eRF3-dependent termination exerts its influence on the subsequent mRNA degradation [16]. Taken together these results suggest a direct link between the termination complex and the mRNA stability.

Both eRF1 and eRF3 are essential for viability of yeast cells and deletion of the C-terminal part of each protein separately lead to lethality (reviewed in [2]). Nonsense *sup45 *mutations have been obtained in the presence of *SUQ5 *suppressor tRNA [17]. However, we have isolated non-lethal nonsense mutations in the *SUP45 *gene of *S. cerevisiae *which lead to decreased level of eRF1 [18]. Nonsense mutations were also obtained in the *SUP35 *gene [19,20]. Here, we show that *sup45 *nonsense and missense mutations have an inhibitory effect on NMD. Our observation that loss of Upf1 suppresses many of the pleiotropic phenotypes caused by mutations in *SUP45 *allowed us to discuss the role of the Upf complex in translation termination.

## Results

### The *sup45 *mutations cause a general decrease in the efficiency of NMD

In previous work, we have isolated non-lethal nonsense and missense mutations in the essential *SUP45 *gene of *S. cerevisiae *which lead to a high level of suppression [18]. Since a direct link between the termination complex and the mRNA stability was proposed, we examined the efficiency of NMD in these mutants by testing whether a decrease in eRF1 level will lead to accumulation of PTC-containing transcripts.

The abundance of the precursor and mature *CYH2 *mRNA levels can be used to monitor NMD, because it has been shown that inefficiently spliced *CYH2 *pre-mRNA containing a premature termination codon is degraded by NMD pathway [21]. In the present paper, we show that the accumulation of *his7-1 *mRNA is affected by NMD pathway. The strain 1B-D1606 in which *sup45 *mutants were obtained, contains nonsense mutations in *HIS7*, *LYS9 *and *TRP1 *genes. As shown in Table 1, sequence analysis of the *his7-1*, *lys9-A21 *and *trp1-289 *alleles identified the presence of single nonsense mutations. It is known that destabilization of mRNAs by NMD depends on the position of the nonsense codon and the presence of a DSE (downstream destabilizing element) downstream of mutation (reviewed in [15]). Indeed, while mRNAs with nonsense codons occurring in the last 20 to 30% of the coding region retain their wild-type decay rates, mRNAs harboring PTC in the first two-third of the gene are subject to degradation by NMD. The *his7-1 *nonsense mutation is located at the beginning of *HIS7 *mRNA and is followed by two putative DSE (Table 1), suggesting that *his7-1 *transcript could be a substrate for NMD.

**Table 1:** Nonsense mutations sequenced in the present work

Mutant allele	Nucleotide substitution		Putative destabilizing motif^a^
		
	Position/ORF size	Codon change^b^	
*his7-1*	229/1659	AAA→TAA	^311^ca**TTGAT**t**TT**aagttgtccaggt**TCGATG**a**TTC**a
*lys9-A21*	605/1341	TTA→TAA	ND
*trp1-289*	403/678	CAG→TAG	^436^gt**TTGAT**t**C**agaagcaggtgggacaggtgaact**T**t**T**g**GAT**tggaac**TCGAT**t**TCT**gactgggttggaagg

^a ^The downstream sequences were searched for the presence of DSE (TGYYGATGYYYYY) [15]. The numbers above the sequences indicate the 5' boundaries of putative DSE (underlined sequences) in the corresponding open reading frame (ORF); ND – not detected. ^b ^Codon changes in the case of each mutation are shown, nucleotide changes are underlined.

**Figure 1:**
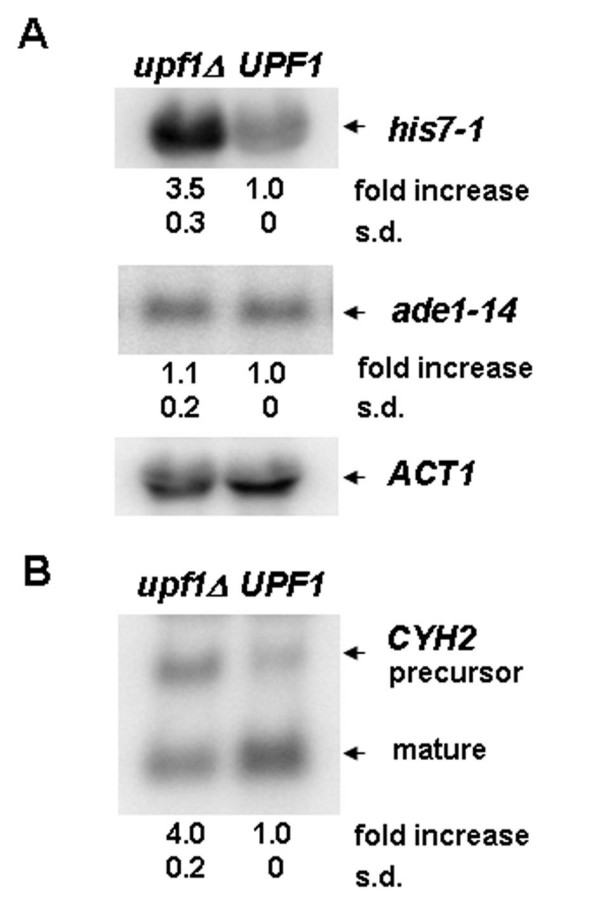
***his7-1 *mRNA accumulated when nonsense-mediated decay is inhibited. **Nothern blotting was used to assess the effect of *UPF1 *deletion on the accumulation of *his7-1 *mRNA. Total RNA was isolated from strain 5B-D1645 (*his7-1 upf1Δ*) transformed with plasmids pRS316 and pRS316/*UPF1*, designated as (*upf1Δ*) and (*UPF1*), respectively. Northern blots were hybridized with radiolabeled *HIS7, ADE1, ACT1 *and *CYH2 *probes. **A**. Representative hybridization signals specific to *his7-1 *mRNA (upper panel), *ade1-14 *mRNA (middle panel) and actin mRNA (*ACT1*) used as an internal control (lower panel) are shown. Numbers indicated under upper and middle panels represent the relative abundance of *his7-1 *and *ade1-14 *mRNA's, respectively, in *upf1Δ *and *UPF1 *strains. (s.d.) – standard deviation. **B**. Accumulation of *CYH2 *precursor mRNA was used to control that NMD is altered in the *upf1Δ *strain. The *CYH2 *probe detects both precursor and mature *CYH2 *mRNA. The fold increase in *CYH2 *precursor/mature mRNA accumulation in *upf1Δ *strain relative to *UPF1 *strain is indicated with the standard deviation (s.d.).

To check if *his7-1 *transcript is affected by NMD, two isogenic strains were constructed harboring the *his7-1 *allele together with *UPF1 *or *upf1*Δ. For this purpose, the strain 5B-D1645 (*his7-1 upf1Δ) *was transformed with pRS316 and pRS316/*UPF1 *plasmids. The mean steady-state level of the *his7-1 *mRNA was 3.5 fold higher in the *upf1*Δ cells than in *UPF1 *cells (Fig. 1, upper panel). However, the level of wild-type *HIS7 *mRNA was not affected by deletion of *UPF1 *(see Additional file 1, figure 1A). The steady-state level of *ade1-14*, another nonsense-containing transcript, did not depend on deletion of *UPF1 *(Fig. 1, middle panel). Therefore, the deletion of *UPF1 *gene affects the *his7-1 *transcript in precisely the same manner that it affects the *CYH2 *pre-mRNA demonstrating that *his7-1 *is a potential substrate for NMD. Earlier it was shown that deletion of *UPF1 *gene promotes suppression of some but not all nonsense mutations [7]. Indeed, we did not detect suppression of *his7-1 *mutation on SC-HIS medium in strain 5B-D1645 bearing *upf1Δ *(see additional file 1 Fig. 1B).

**Figure 2:**
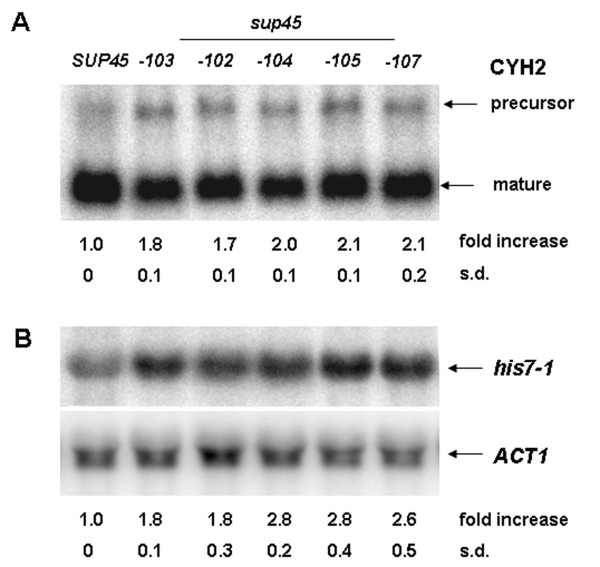
**Nonsense or missense alleles of *SUP45 *affect accumulation of *his7-1 *mRNA and *CYH2 *pre-mRNA**. Northern blots were prepared with total RNA from wild-type strain 1B-D1606 (*SUP45*) and its *sup45 *mutant derivatives. Blots were hybridized with DNA probes that detected the *his7-1, CYH2 *and *ACT1 *transcripts. For each mutant the average *CYH2 *pre-mRNA/mRNA ratio (**A**) and the abundance of *his7-1 *mRNA (**B**) relative to the wild-type strain are shown with the standard deviation (s.d.). Following *sup45 *mutations were tested: 102, 104, 105, 107 (nonsense) and 103 (missense).

We studied NMD efficiency in *sup45 *mutant strains by examination of *CYH2 *and *his7-1 *mRNA levels. First, we have shown that the mRNA level of wild-type *HIS7 *mRNA in *sup45-n *mutants and *upf1Δ *mutants is not changed (see additional file 1 Fig. 1B). Next, we used strains harboring nonsense mutations *sup45*- *102, 104, 105, 107*, which lead to decrease of eRF1 full-length protein level [18] and the *sup45*-*103 *(L21S) missense-mutation [22]. Total RNA was isolated from mutants and analyzed by Northern blots using probes specific for *CYH2 *and *his7-1 *mRNA. Strains bearing *sup45-n *mutations had a significantly increased *CYH2 *pre-mRNA/RNA ratio (1.7 ± 0.1 to 2.1 ± 0.2) compared with that of wild-type *SUP45 *strain (ratio of 1). A similar increase was also observed for *sup45-103 *missense mutant (1.8 ± 0.1) (Fig. 2A). Similarly, the relative abundance of *his7-1 *mRNA in the *sup45-n *mutant strains, normalized using *ACT1 *mRNA, ranged from 1.8 ± 0.3 to 2.8 ± 0.4 compared with that of wild-type *SUP45 *strain (ratio of 1) (Fig. 2B). The strain harboring missense mutation *sup45-103 *was also characterized by the accumulation of the *his7-1 *transcript (1.8 ± 0.1). This effect was weaker than that observed for *UPF1 *deletion which causes 4 and 3.5 fold increases in the *CYH2 *pre-mRNA/RNA ratio and the accumulation of *his7-1 *mRNA, respectively (Fig. 1), however it appears specific for NMD substrates since the amounts of other nonsense-containing transcripts (*ade1-14 *and *lys9-A21*) were not significantly changed in *sup45 *mutants (see Additional file 2). We also observed a significant increase of the *CYH2 *pre-mRNA/RNA ratio and of *his7-1 *mRNA level in strains bearing *sup35 *nonsense and missense mutations (data not shown). Taken together, these results demonstrate that both nonsense and missense mutations in *SUP45 *decrease the efficiency of mRNA degradation by NMD thus leading to accumulation of mRNAs containing PTCs.

### Increased viability of *sup45 *nonsense mutants in the absence of *UPF1*

Previously, we have shown that *sup45 *nonsense mutants are viable in different genetic backgrounds [18]. However, the efficiency of plasmid shuffle was significantly lower in the case of mutant *sup45-n *alleles compared to plasmid bearing wild-type *SUP45 *gene [18] indicating that *sup45-n *mutations imperfectly replace *SUP45*. To assess the effects of double *sup45 upf1 *mutations on viability of corresponding strains we performed plasmid shuffle analysis using strains bearing single *sup45 *mutations or *sup45 *in combination with *upf1Δ *(see Materials and Methods).

**Figure 3:**
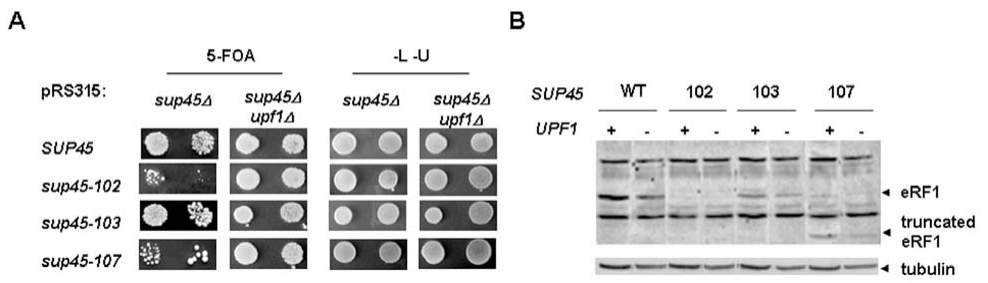
**Deletion of *UPF1 *gene leads to increased viability of *sup45 *nonsense mutants. (A)**. Strains 1A-D1628 (*sup45Δ*) and 1-1A-D1628 (*sup45Δ upf1Δ*) all containing *SUP45 *deletion and pRS316/*SUP45 *plasmid were transformed with pRS315/*SUP45-LEU2 *plasmids carrying different *sup45 *mutant alleles. The growth of the transformants was tested by plating 10^0^, and 10^-1 ^serial dilutions of overnight cultures (*left *to *right*) onto 5-FOA plates. The extent of cell growth on 5-FOA plates indicates the ability of the *sup45 *mutant alleles to support cell growth in the presence and absence of *UPF1 *gene. The same serially diluted cultures were also spotted on synthetic complete plates lacking leucine and uracil (-L -U) to estimate the total number of cells analyzed. (**B**). Level of eRF1 protein in the clones selected on 5-FOA medium was analyzed by western blot. Tubulin was used as a loading control. Following *sup45 *mutations were tested: 102, 107 (nonsense) and 103 (missense).

Two nonsense mutations resulting in different stop codons (*sup45-102 *(UAA) and *sup45-107 *(UGA)) and one missense mutation (*sup45-103) *were used to perform plasmid shuffle experiments. Two isogenic yeast strains 1A-D1628 (*sup45Δ *pRS316/*SUP45*) and 1-1A-D1628 (*sup45Δ upf1Δ *pRS316/*SUP45*) were transformed with the pRS315 plasmids bearing the wild-type *SUP45 *gene or different *sup45 *mutations. Transformants were then subjected to plasmid shuffle analysis to verify whether strains containing the *sup45 *alleles could lose the plasmid carrying the wild-type gene. In the *sup45Δ *strain, all transformants were able to grow in the presence of 5-FOA, indicating that all tested mutations can replace wild-type *SUP45*. However, as previously described [18], plasmid shuffle was less efficient with *sup45 *mutations than with wild-type *SUP45*. Surprisingly, introduction of *upf1Δ *mutation lead to increased viability of *sup45 *mutants (Fig. 3, 5-FOA). We did not observe difference in growth between wild-type and *sup45 *mutants on medium selective for both plasmids (Fig. 3, -L-U). In order to check that deletion of *UPF1 *does not lead to higher production of eRF1 protein in double *sup45 upf1Δ *mutants which could explain the increased viability of these mutants, we analyzed eRF1 protein level by western blot. As shown in figure 3B, deletion of *UPF1 *does not affect the level of eRF1 protein in *sup45 *mutants.

The same experiment was repeated using two other strains, 3b-D1658 (*sup45Δ *pRS316/*SUP45*) and 3v-D1658 (*sup45Δ upf1Δ *pRS316/*SUP45*). These strains were transformed with the pRS315 plasmids bearing the wild-type *SUP45 *gene or different *sup45 *mutations, Leu^+ ^Ura^+ ^transformants were patched on 5-FOA medium to select against pRS316/*SUP45 *plasmid (see Additional file 3, Fig. 3AB). The same results were obtained. Again, introduction of *upf1Δ *mutation leads to increased viability of *sup45 *mutants (see Additional file 3, Fig. 3AB). Western blot analyses showed that deletion of *UPF1 *did not affect levels of eRF1 and eRF3 proteins in *sup45 *mutants (see additional file 3 Fig. 3C). Thus, deletion of *UPF1 *confers selective survival advantages to *sup45 *nonsense mutants.

### Deletion of the *UPF1 *gene suppresses several *sup45 *phenotypes

**Figure 4:**
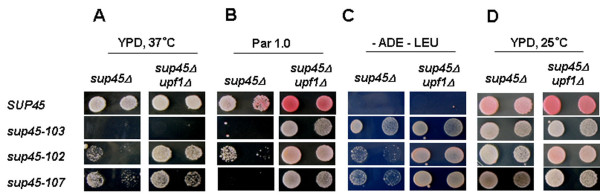
**Deletion of the *UPF1 *gene suppresses several *sup45 *phenotypes**. The growth of the transformants of two isogenic strains, 1A-D1628 (*sup45Δ*) and 1-1A-D1628 (*sup45Δ upf1Δ*), selected on 5-FOA medium (Fig. 3A) was tested by plating 10^0^, and 10^-1 ^serial dilutions of overnight cultures (*left *to *right*) onto YPD plates at 37°C (**A)**, onto YPD plates with 1 mg/ml paromomycin (**B) **and onto synthetic complete -adenine -leucine plates (**C**)**. **The same serially diluted cultures were also spotted on YPD plates at 25°C (**D) **to estimate the total number of cells analyzed. Following *sup45 *mutations were tested: 102, 107 (nonsense) and 103 (missense).

It is known that mutations in the *SUP45 *gene lead to suppression of nonsense mutations and also to many phenotypic changes including high or low temperature sensitivity, respiratory efficiency and sensitivity to aminoglycoside antibiotics such as paromomycin (reviewed in [2]). It has been previously reported that loss the *UPF1 *gene results in the suppression of some but not all of nonsense mutations. In addition, deletion of *UPF1 *does not confer sensitivity to paromomycin [7], an aminoglycoside antibiotic that induces translational misreading. We therefore compared phenotypes of single *sup45 *and double *sup45 upf1Δ *mutants, previously obtained by plasmid shuffle, for suppression efficiency, temperature sensitivity and sensitivity to paromomycin. As previously described [18,22], nonsense *sup45-102 *and *sup45-107 *and missense *sup45-103 *mutations are temperature sensitive. We observed that deletion of *UPF1 *suppressed temperature sensitivity of both *sup45-102 *and *sup45-107 *nonsense mutants in rich medium. However *UPF1 *deletion did not suppress temperature sensitivity of *sup45-103 *missense mutant (Fig. 4A). The observed disparity in effect of *UPF1 *deletion on temperature sensitivity of nonsense and missense mutants can be connected with different nature of temperature sensitivity in the case of decreased level of eRF1 compared with mutated eRF1. Loss of Upf1 also restored growth of all *sup45 *mutants on paromomycin media (Fig. 4B). In addition, deletion of *UPF1 *had an allosuppressor effect on suppression of *ade1-14 *mutation by *sup45 *mutations. However deletion of *UPF1 *alone, in the presence of wild-type copy of the *SUP45 *gene had no suppressor effect on *ade1-14 *mutation (Fig. 4C). On YPD medium, all transformants grew indicating that they retained their growth capacity on rich medium (Fig. 4D). Therefore the analysis of *sup45 upf1Δ *double mutants shows that loss of Upf1 not only affects viability of *sup45 *mutants but also suppresses several *sup45 *phenotypes.

### Defects of NMD in double mutants *sup45 upf1*Δ

**Figure 5:**
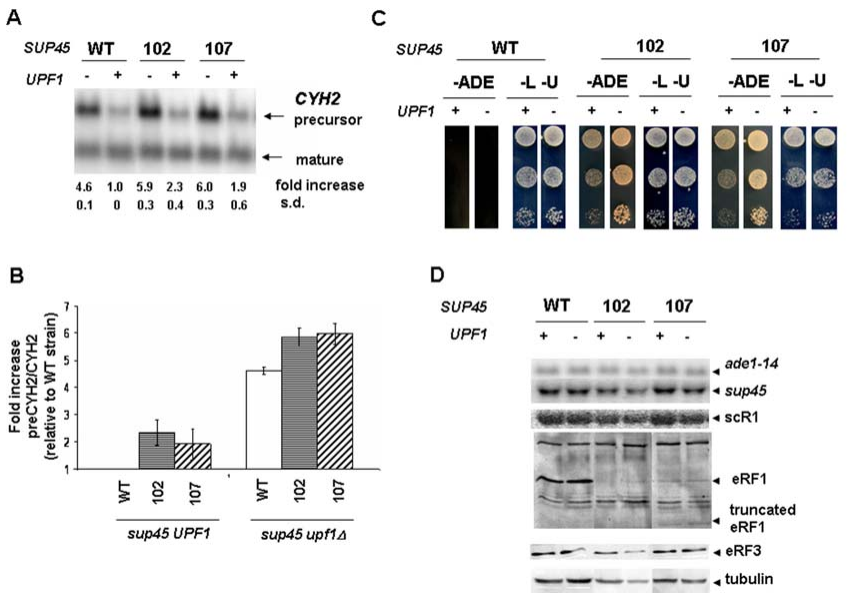
**Double mutants *sup45 upf1Δ *are characterized by defects of NMD**. **A**. Representative hybridization signals specific to precursor and mature forms of *CYH2*. Total RNA was isolated from strain 3v-D1658 (*sup45Δ upf1Δ *pRS315/*SUP45*) and its derivates (*sup45Δ upf1Δ *pRS315/*sup45-n*) transformed with pRS316 and pRS316/*UPF1 *plasmids, designated as (*UPF1 *-) and (*UPF1 *+), respectively. The Northern blots were hybridized with radiolabeled *CYH2 *probe. The *CYH2 *precursor/mature ratio in wild-type strain was set as 1.0. **B**. The fold increase in *CYH2 *precursor/mature mRNA accumulation measured in the same strains as in panel A are represented relative to such in wild-type strain. **C**. The same transformants as in panel A were tested by plating 10^0^, 10^-1^and 10^-2 ^serial dilutions of overnight cultures (*left *to *right*) on synthetic complete plates lacking adenine and incubated 5 days at 25°C. The same serially diluted cultures were also spotted on plates lacking leucine and uracil (-L -U) to estimate the total number of cells analyzed. **D**. Norhern blots prepared with total RNA from the same transformants as in panel A were hybridized with radiolabeled probes, detecting *ade1-14*, *SUP45 *and scR1 mRNA (scR1 was used as a control). eRF1 and eRF3 protein levels in the same transformants were analyzed by western blot. Tubulin was used as a loading control. WT – wild type, 102 – *sup45-102 *(nonsense), 107 – *sup45-107 *(nonsense).

We have previously shown that *sup45 *mutants affect NMD and that *UPF1 *deletion suppresses several *sup45 *phenotypes. Therefore, we next examined if *UPF1 *deletion would have an additional effect on NMD in *sup45 *mutants. For this purpose, we compared accumulation of *CYH2 *precursor mRNA in single *sup45 *mutants and *sup45 upf1Δ *double mutants. To this end, we transformed strain 3v-D1658 (*sup45Δ upf1Δ *pRS315/*SUP45*) and its derivates (*sup45Δ upf1Δ *pRS315/*sup45-n*) with plasmids pRS316 or pRS316/*UPF1*. In the presence of *upf1Δ *and wild-type *SUP45*, accumulation of *CYH2 *precursor increased by 4.6 fold. We found that the presence of *sup45 *mutations increased the ratio of pre*CYH2 *to mature *CYH2 *mRNA by 1.9 to 2.3-fold (Fig. 5A,B), in agreement with results obtained previously in a different genetic background (Fig. 2A). Combination of *sup45-n *mutations and *upf1Δ *slightly but reproducibly increased the ratio of pre*CYH2 *to mature *CYH2 *mRNA from 4.6 to 6.0 fold. Therefore, these results indicate that the combination of *sup45-n *and *upf1 *mutations together increased accumulation of *CYH2 *precursor more than either single one.

To compare the effects of single *sup45 *mutations and the *sup45 upf1Δ *double mutations on the efficiency of suppression, we replica plated the same transformants that were used for Nothern blots analysis (Fig. 5A) on adenine deprived medium. As shown in another genetic background (Fig. 4C), deletion of *UPF1 *does not promote suppression of *ade1-14 *mutation (Fig. 5C) and the combined effects of *sup45 *mutations and *upf1Δ *promote increase in the suppression efficiency compared with *sup45 *mutations alone. As shown in figure 5D, allosuppression of *ade1-14 *by deletion of *UPF1 *is not the result of stabilization of *ade1-14 *mRNA in double *sup45 upf1Δ *mutants. We also showed that deletion of *UPF1 *does not affect *sup45-n *mRNAs and eRF1 as well as eRF3 protein levels (Fig. 5D).

### Deletion of either *UPF2 *or *UPF3 *increases viability of *sup45 *nonsense mutants

**Figure 6:**
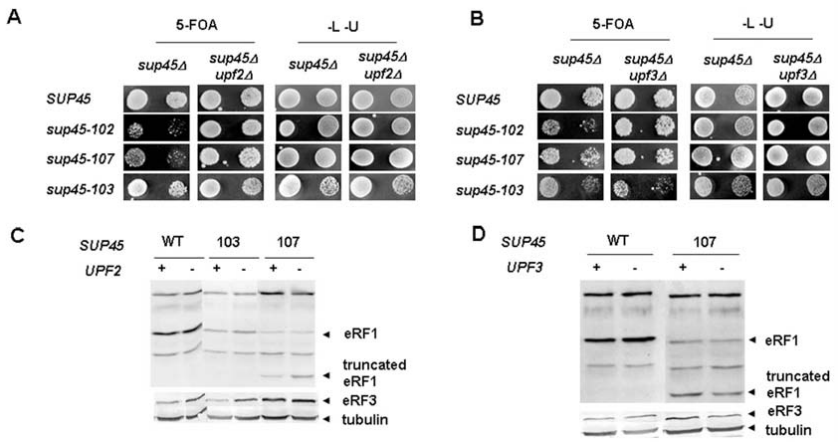
**Deletion either *UPF2 *or *UPF3 *gene leads to increased viability of *sup45 *nonsense mutants**. Strains 4a-D1659 (*sup45Δ*) and 4b-D1659 (*sup45Δ upf2Δ*) **(A)**, 18a-D1660 (*sup45Δ*) and 3a-D1660 (*sup45Δ upf3Δ*) **(B)**, all containing *SUP45 *deletion and pRS316/*SUP45 *plasmid were transformed with pRS315/*sup45-n-LEU2 *plasmids carrying different *sup45 *mutant alleles. The growth of the transformants was tested by plating 10^0^, and 10^1 ^serial dilutions of overnight cultures (*left *to *right*) onto 5-FOA plates. The extent of cell growth on 5-FOA plates indicates the ability of the *sup45 *mutant alleles to support cell growth in the presence and absence of *UPF2 *or *UPF3 *genes. The same serially diluted cultures were also spotted on synthetic complete plates lacking leucine and uracil to estimate the total number of cells analyzed. (**C, D**) eRF1 and eRF3 protein levels in the same transformants as in panels A and B were analyzed by western blot. Tubulin was used as a loading control. Following *sup45 *mutations were tested: 102, 107 (nonsense) and 103 (missense).

It has been described that functional NMD requires not only Upf1 but also Upf2 and Upf3 [7,9-11,23-25]. To test whether *UPF2 *or *UPF3 *deletion will have the same effect on viability of *sup45 *nonsense mutants as deletion of *UPF1*, we transformed strains 4a-D1659 (*sup45Δ*), 4b-D1659 (*sup45Δ upf2Δ*), 18a-D1660 (*sup45Δ*) and 3a-D1660 (*sup45Δ upf3Δ*), all containing pRS316/*SUP45 *[*URA3*] plasmid, with pRS315/*sup45-n *[*LEU2*] plasmids carrying *sup45-102 *or *sup45-107 *mutant alleles. Leu^+ ^Ura^+ ^transformants were selected and plated on leucine deprived medium containing 5-FOA to select against the pRS316/*SUP45 *[*URA3*] plasmid. Combining either *UPF2 *or *UPF3 *deletion with *sup45 *mutations results in an enhanced viability of double mutants (Fig. 6AB). Western blot analysis has shown, as in case of *UPF1 *deletion, that the level of eRF1 and eRF3 proteins was not dependent on the presence of functional *UPF2 *or *UPF3 *genes (Fig. 6CD). These results demonstrate that deletion of *UPF2 *or *UPF3 *as well as deletion of *UPF1*, all essential components of NMD pathway, leads to increase viability of *sup45-n *mutant strains.

## Discussion

In the present work, we have shown that nonsense and missense mutations in the *SUP45 *gene lead to stabilization of PTC-containing mRNAs degraded by NMD. This is the first demonstration that *sup45 *mutations do not only change translation fidelity but also acts by causing a change in mRNA stability.

The *CYH2 *pre-mRNA which contains a premature termination codon was previously shown to be degraded by NMD pathway [21]. We also identified that NMD affects accumulation of *his7-1 *mRNA. A single A→T mutation in this allele leads to change of codon 229 for UAA. In addition, two imperfect putative DSE are found downstream of this premature stop codon. Using a *upf1Δ *strain, we demonstrated that the *his7-1 *transcript is possibly under control of NMD pathway. In order to answer if changes in transcription of *HIS7 *gene could account for accumulation of *his7-1 *mRNA, we examined the mRNA level of wild-type *HIS7 *mRNA in *sup45-n *mutants and *upf1Δ *mutants. Deletion of *upf1 *as well as *sup45 *mutations leads to accumulation of *his7-1 *mRNA but do not affect mRNA level of wild-type *HIS7 *mRNA. Accordingly, a genome-wide analysis performed in strains depleted for NMD showed that wild-type *HIS7 *mRNA (and also *ADE1 *and *LYS9 *mRNAs) is not affected in strains deleted for *upf1 *[26].

Here, we demonstrate that accumulation of *his7-1 *and *CYH2 *precursor mRNAs in cells bearing *sup45 *mutations was much higher than that observed in the wild-type strain. But, *sup45 *mutations do not promote accumulation of other nonsense-containing transcripts, such as *ade1-14 *or *lys9-A21*, despite efficient suppression of these mutations, as well as *his7-1 *[18,22]. This result indicates that simply increasing read-through efficiency does not result in a general increase in the abundance of PTC-containing mRNAs but that *sup45 *mutations specifically affect PTC-containing mRNA subjected to NMD. We observed that the abundance of *his7-1 *and *CYH2 *precursor mRNAs in cells bearing *sup45 *mutations was lower compared to those in the *upf1Δ *strain. This difference between *upf1Δ *and *sup45 *mutants could be explained by the complete absence of Upf1 protein in the *upf1Δ *strain leading to complete inactivation of NMD and by the presence of some functional eRF1 protein in *sup45 *mutants which is necessary for cell viability [18,22]. Indeed, we previously reported that in the case of *sup45-n*, the level of eRF1 is decreased compared to wild-type and in the case of *sup45 *missense mutants the level of eRF1 is unchanged but its functionality is altered. These results demonstrate that eRF1 participates in NMD.

Recently, it was shown the importance of the second translation termination factor (eRF3) for NMD and interaction of both eRF1 and eRF3 with Upf proteins. Upf1 protein interacts with the polypeptide release factors eRF3 and eRF1 while they are still present in the ribosome-bound termination complex, providing a direct link between the termination complex and the NMD machinery [12,13]. Both Upf2 and Upf3 interact with eRF3, but not with eRF1; and Upf2, Upf3 and eRF1 compete with each other *in vitro *for binding to eRF3 [12,13]. eRF3 also interacts with poly(A)-binding protein (PABP) [27,28], furthermore, eRF3 regulates the initiation of normal mRNA decay at the poly(A) tail-shortening step through the interaction with PABP [29]. Thus, eRF3 can mediate normal and nonsense-mediated mRNA decay through its association with Pab1 and Upf1 and therefore was proposed as a key mediator between translation termination and NMD [16]. Moreover, it was previously shown that a weak translation termination due to [*PSI*^+^] (a prion form of eRF3) antagonizes the effects of NMD [30]. A first indication for a link between translation termination and NMD came from observations that decay of PTC-containing mRNAs can be antagonized by tRNAs that suppress termination [31]. Data have shown that normal termination is distinct from premature termination and this difference is dependent upon the presence of Upf1 at the premature termination codon [32]. Our results together with data about eRF1-Upf1 interaction [12,13] demonstrate that eRF1 as well as eRF3 is an essential factor linking translation termination and NMD. Recognition of stop codons is a common event necessary for the two processes. Since it is established that eRF1 plays a crucial role in translation termination by directly recognizing stop codons (reviewed in [2]), eRF1 could have an identical function in NMD by recognition of PTC.

We observed that the combination of *upf1Δ *and *sup45-n *mutations leads to an increase in *CYH2 *precursor mRNA abundance that was higher than in *upf1Δ *and *sup45-n *single mutants. A similar additive effect on stabilization of nonsense-containing mRNA was shown for combination of *upf1Δ *and [*PSI*^+^] [30]. Therefore, a possible explanation for this additive effect of *upf1Δ *and *sup45-n *mutations could be that eRF1 is required for both normal and nonsense-mediated mRNA decay, as it was shown for eRF3 [16].

It has been shown that mutation in eRF3 which impairs eRF3 binding to eRF1 affected mRNA decay [16]. In the present paper, we show that the missense mutation *sup45-103 *(L21S) alters degradation of PTC-containing mRNAs by NMD. However, we have previously shown that this mutation does not affect the eRF1-eRF3 interaction [22], indicating that this allele has an inhibitory effect on NMD that is independent on eRF1-eRF3 binding. This result demonstrates that eRF1 mutation affecting PTC-containing mRNA decay by NMD does not obligatory alters the eRF1-eRF3 interaction.

A role for the Upf1 protein, essential for NMD, in translation termination first became evident when a set of mutations were isolated in the *UPF1 *gene that separated the mRNA decay function from its activity in modulating premature termination [33,34]. Subsequent studies have shown that deletion of either *UPF2 *or *UPF3 *can also lead to a nonsense suppression phenotype [7,9,11,13,35]. In addition, it was shown that *upf1Δ *mutation causes a general decrease in the efficiency of translation termination at UAG, UAA, and UGA stop codons [30].

In this work, we have shown that deletion of *UPF1 *does not affect *ade1-14 *mRNA level, but results in allosuppression of *ade1-14 *mutation in *sup45 *nonsense mutants therefore revealing that deletion of *UPF1 *has a synergistic effect with *sup45-n *mutants. Similar allosuppressor effect has also been shown for deletion of *UPF1 *in combination with [*PSI*^+^] [30,36]. Based on this additive effect, Keeling et al. [30] proposed that *upf1Δ *mutation and [*PSI*^+^] influence the termination process in distinct ways. Our results suggest that this could be also the case for *upf1Δ *and *sup45 *mutations.

We found that deletion of the *UPF1 *gene affects several other *sup45 *phenotypes, such as temperature sensitivity, paromomycin sensitivity and viability of *sup45 *mutants. It is known that deletion of *UPF1 *gene in yeast does not cause any detectable phenotypic effects except respiratory deficiency [37] and nonsense suppression [7,9,13,33-35]. Also telomere length is affected by deletions of *UPF1*-3 genes [38]. How *UPF1 *deletion could affect *sup45 *phenotypes? We can not exclude an indirect effect of *UPF1 *deletion on *sup45 *phenotypes. It has been reported that NMD controls the mRNA levels of several hundred of wild-type genes [24,26]. One can hypothesize that depletion of Upf1 could affect the expression of some translation apparatus components (*e.g*. tRNA genes) which themselves influence the viability of *sup45 *mutants. Indeed, the presence of *SUQ5 *mutation, a mutant suppressor tRNA^Ser^, increases the viability of *sup45-n *mutants [18]. Alternatively, since inactivation of the NMD pathway by *upf1Δ *mutation does not increase the steady-state levels of wild-type and mutant *SUP45 *mRNAs and does not cause a change in the amount of eRF1 protein, we propose that the effect of NMD on *sup45 *phenotypes is probably *via *a change in the stoichiometry of factors involved in translation termination and NMD. In contrast to mammals, Upf proteins of *S. cerevisiae *are present at very low intracellular concentrations [39]. Considering that in *sup45-102 *and *sup45-107 *nonsense mutants the amount of eRF1 was estimated as 8% and 17% of wild-type level, respectively [18], in mutant cells eRF1 and Upf1 proteins are probably present in stoichiometric amounts. Possibly, in wild-type cells, Upf1 is not preventing normal termination because its amount is ten times lower than eRF1, but in the case of their presence in stoichiometric amounts in *sup45 *nonsense mutants binding of Upf1 to eRF1 could result in a defective complex formation that blocks termination. This hypothesis is supported by finding that viability of *sup45 *nonsense mutants depends also on Upf2 or Upf3 proteins. There is a possibility that effect of Upf2 or Upf3 depletion is indirect and is under control of Upf1. It was shown that in mammalian cells a depletion of Upf2 or Upf3 reduces the amount of the phosphorylated form of Upf1 possibly preventing Upf1 dissociation from eRF3 and eRF1 [40]. Phosphorylation of Upf1 and Upf2 was also shown in *S. cerevisiae *[41,42], an indication that this mechanism might operate in yeast cells as well.

From recent studies, it appears more and more clear that translation termination and mRNA stability are intimately linked and our results demonstrate that eRF1 is also an essential factor linking these two processes.

## Conclusion

In the present work, we have shown hat nonsense and missense mutations in *SUP45 *gene lead to stabilization of *CYH2*, a PTC-containing pre-mRNA degraded by NMD, and to accumulation of *his7-1 *mRNA. At the same time, *sup45 *mutations do not promote accumulation of other nonsense-containing transcripts, such as *ade1-14 *or *lys9-A21*, despite efficient suppression of these mutations. Thus *sup45 *mutations specifically affect PTC-containing mRNA subjected to NMD. Deletion of *UPF1 *results in allosuppression of *ade1-14 *mutation in *sup45 *nonsense mutants and leads to an increase in *CYH2 *pre-mRNA abundance therefore revealing that deletion of *UPF1 *has a synergistic effect with *sup45-n *mutants. This is the first demonstration that *sup45 *mutations do not only change translation fidelity but also acts by causing a change in mRNA stability.

Models explaining increased viability of *sup45 *nonsense mutants in the absence of Upf1, Upf2 or Upf3 proteins are proposed. First, the depletion of Upf1 could affect the expression of some translation apparatus components (*e.g*. tRNA genes) which themselves influence the viability of *sup45 *mutants. Second, a change in the stoichiometry of factors involved in translation termination and NMD provides the effect of NMD on *sup45 *phenotypes.

## Methods

### Yeast strains, plasmids and growth conditions

The *S. cerevisiae *strains used in this study are listed in Table 2. Previously characterized *sup45 *mutations [18,22] were used in this study, among them the following nonsense mutations (*sup45-n*): *sup45-102 *(53 Tyr→TAA); *sup45-104 *(283 Leu→TAA); *sup45-105 *(*385 *Glu→TAA); *sup45-107 *(317 Leu→TGA); as well as missense mutation (*sup45-m*): *sup45-103 *(Leu21Ser). All *sup45 *mutations were selected by spontaneous reversions of two nonsense mutations, *his7-1 *and *lys9-A21 *in strain 1B-D1606. Strains Y06214, Y01905, Y04702 are from Euroscarf; other strains were obtained during this study. Strain 5B-D1645 was recovered from the meiotic progeny of D1645, obtained by mating Y06214 and 16A-D1608. Strains D1658, D1659, D1660 were generated by mating of strains Y06214, Y01905, Y04702 with 1A-D1628. Tetrads were dissected and segregants were used for further study. Yeast strain 1-1A-D1628 was generated by using a one-step gene replacement method. The *UPF1 *gene was deleted by the removal of the entire open reading frame and the insertion of the kanMX gene by using PCR-based gene deletion approach [43] with plasmid pFA6a-kanMX. The following primers were used for PCR: F1 (AATATACTTTTTATATTACATCAATCATTGTCATTATCAACGGATCCCCGGGTTAATTAA) and R1 (AAGCCAAGTTTAACATTTTATTTTAACAGGGTTCACCGAAGAATTCGAGCTCGTTTAAAC). Yeast strain 1A-D1628 was transformed with the fragment generated by PCR. Kan^R ^transformants were screened by PCR. Yeast strains were grown either in standard rich or synthetic culture medium [44] at 25°C. Transformants were grown in the media selective for plasmid maintenance (SC-Trp, SC-Leu, SC-Ura). Suppression of nonsense mutations was estimated by growth at 25°C on synthetic media lacking the corresponding amino acids. For plasmid shuffle, selective medium containing 1 mg/ml 5-fluoroorotic acid (5-FOA, Sigma) was used. Yeast transformation was performed as described [45]. Plasmid pRS316/*UPF1 *contains *UPF1 *gene under its own promoter [46].

**Table 2:** Strains used in this study

Strain	Genotype
1B-D1606	*MATα ade1-14(UGA) his7-1(UAA) trp1-289(UAG) lys9-A21(UAA) ura3-52 leu2-3,112*
5B-D1645	*MATa ade1-14(UGA) his7-1(UAA) trp1 ura3 upf1::kanMX4*
16A-D1608	*MATa ade1-14 his7-1 lys2-87 met13-A1 thr4-B15 trp1 ura3-52 leu2-3,112 SUP35::TRP1*
Y06214	*MATa his3Δ1 ura3Δ0 leu2Δ0 met15Δ0 upf1::kanMX4*
Y01905	*MATa his3Δ1 ura3Δ0 leu2Δ0 met15Δ0 upf2::kanMX4*
Y04702	*MATa his3Δ1 ura3Δ0 leu2Δ0 met15Δ0 upf3::kanMX4*
3b-D1658	*MATa ade1-14 his3 leu2 lys2 ura3 sup45::HIS3 *[pRS316/*SUP45*]
3v-D1658	*MATα ade1-14 his3 leu2 lys2 ura3 sup45::HIS3 *[pRS316/*SUP45*]* upf1::kanMX4*
4a-D1659	*MATα ade1-14 his3 leu2 lys2 ura3 sup45::HIS3 *[pRS316/*SUP45*]
4b-D1659	*MATa ade1-14 his3 leu2 lys2 met15Δ ura3 sup45::HIS3 *[pRS316/*SUP45*] *upf2::kanMX4*
18a-D1660	*MATa ade1-14 his3 leu2 ura3 lys2 sup45::HIS3 *[pRS316/*SUP45*]
3a-D1660	*MAT α ade1-14 his 3 leu2 ura3 lys2 sup45::HIS3 *[pRS316/*SUP45*] *upf3::kanMX4*
1A-D1628	*MATα ade1-14 his3 lys2 ura3-52 leu2-3,112 trp1 sup45::HIS3 *[pRS315/*SUP45*]
1-1A-D1628	*MATα ade1-14 his3 lys2 ura3-52 leu2-3,112 trp1 sup45::HIS3 *[pRS315/*SUP45*] *upf1::kanMX*

### Plasmid shuffle

The haploid *SUP45::HIS3 *[*CEN URA3 SUP45*] and *SUP45::HIS3 UPF::kanMX4 *[*CEN URA3 SUP45*] strains were used in "plasmid shuffle". These strains were transformed with [*CEN LEU2 sup45*] plasmids. Transformants, selected on -Ura-Leu medium, were velveteen replica plated onto 5-FOA medium, which counterselects against *URA3 *plasmids [47]. Growth was also assayed using serial dilutions of overnight cultures with OD_600 _= 1. Serially (10-fold) diluted yeast cell cultures were spotted on plates containing 5-FOA to determine the ability of the *sup45 *mutant alleles to support cell growth in the presence and absence of any one of three *UPF *genes. The wild-type yeast *SUP45 *gene carried on the *URA3 *plasmid eliminates because 5-FOA is toxic to cells expressing the *URA3 *gene. The same serially diluted cultures were also spotted on plates lacking leucine and uracil to estimate the total number of cells analyzed.

### Sequencing of the alleles *his7-1*, *lys9-A21 *and *trp1-289*

Yeast DNA was prepared using genomic DNA purification Kit (Promega). DNA fragments, corresponding to ORFs were amplified with the following primers:

*HIS7 *(202 GGCAGCTATTGAAGTAGCAGTATCCAG and

203 CCCTACTGACACCACCAATAATACAACC),

*LYS9 *(192 CAGCAATAGATGATAGAAAGTAGCACAG and

193 CAAGCTTCAGGAACTACACTCTC),

*TRP1 *(188 GAGGGAGGGCATTGGTGACTATTG and

189 GCACAAACAATACTTAAATAAATACTACTC).

For each allele at least two independent PCR-products were sequenced using the following primers:

*HIS7 *(195 GATGTACGTACTAATGACCAAGGTG and

196 GTTACTTCATCCGCACCCTGTTGG),

*LYS9 *(197 GCTAAATACTGGAAAGACGGAAAG and

198 GATATCTCAAAGTACCTCTAATGACCG),

*TRP1 *(177 GTCTGTTATTAATTTCACAGGTAGTTC).

### Analysis of mRNA steady-state levels

Total RNA was prepared by hot-phenol extraction method from yeast culture grown in YPD medium to log phase OD_600 _= 0.5-0.8 as described [44]. Five micrograms of each RNA sample were separated on a 1.2% agarose gel, containing 3% formaldehyde and transferred to nylon membrane, Z-probe (Bio-Rad). *SUP45*, *HIS7, CYH2 *and *ACT1 *transcripts were detected using gene-specific ^32^P-radiolabelled DNA probes. Radioactive signals were directly detected and quantified by STORM Phosphor Imager system (Molecular dynamics, USA).

Probes were synthesized using following oligos:

82 CATTTCGGCTTGTCTCC and 83 TCTGGCATCTAGTGATTAAATTC (for *SUP45*), 195 GATGTACGTACTAATGACCAAGGTG and 196 GTTACTTCATCCGCACCCTGTTGG (for *HIS7*), 186 GACTAGAAAGCACAGAGGTCACGTC and 187 GACTAGAAAGCACAGAGGTCACGTC (for *CYH2*), 175 CGAAGACTGAACTGGACGGTATATTG and 176 CCCTGTCAATGTTTCATAAGCCTC for *ADE1*, 192 CAGCAATAGATGATAGAAAGTAGCACAG and 193 CAAGCTTCAGGAACTACACTCTC (for *LYS9*), 215 AGGCTGTAATGGCTTTCTGGTGGGATGGGA and 216 GATATGTGCTATCCCGGCCGCCTCCATCAC (for scR1).

*Hind*III-*Xba*I fragment of plasmid pSK/actin was used as a probe for *ACT1 *(M. Vedel, Institut of M. Curie, Paris).

### Protein analysis

Protein isolation, SDS-PAGE electrophoresis, and western blotting were performed as described previously [18]. Antibodies specific to eRF1 and eRF3 were described previously [18,20], monoclonal anti-α-tubulin antibodies were described before [48]. eRF1 and eRF3 and α-tubulin signals were detected using alkaline-phosphatase-coupled anti-rabbit immunoglobulin G secondary antibodies (Jackson) (for eRF1 and eRF3) or alcaline-phosphatase-coupled anti-mouse immunoglobulin G secondary antibodies (Jackson) (for tubulin) by Amersham ECF system (Amersham Pharmacia Biotech). Signals were quantified with STORM 840 Phosphor-Imager (Molecular dynamics, USA) and ImageQuantNT 5.2 software.

## Authors' contributions

SC, carried out the molecular genetic studies and drafted the manuscript, VG, constructed all strains with *UPF *gene deletions, SM and CLG participated in the molecular genetic studies, GZ, designed the study, performed plasmid shuffle analysis and wrote the manuscript. All authors read and approved the final manuscript.

## Supplementary Material

Additional file 1 **A. Deletion of *UPF1 *and mutation of *SUP45 *do not affect the wild-type *HIS7 *mRNA level**. Total RNA was isolated from strain 3v-D1658 (*sup45Δ upf1Δ *pRS315/*SUP45*) and its derivative (*sup45Δ upf1Δ *pRS315/*sup45-102*) transformed with pRS316 and pRS316/*UPF1*. Blots were hybridised with DNA probes that detected the wild-type *HIS7 *and *ACT1 *transcripts (*ACT1 *was used as a loading control). The fold increase in *HIS7/ACT1* mRNA accumulation relative to such in wild-type strain is shown. The *HIS7/ACT1* ratio in wild-type strain was set as 1.0. **B. Deletion of *UPF1 *does not suppress *his7-1 *mutation**. Strain 5B-D1645 (*his7-1 upf1Δ*) transformed with plasmids pRS316 and pRS316/*UPF1*, designated as (*UPF1 *-) and (*UPF1 *+), respectively. The growth of the transformants was tested by plating 10^0^, and 10^1 ^serial dilutions of overnight cultures on a synthetic complete medium without histidine (-HIS). This strain bears *his7-1 *mutation and is unable to grow on such medium except if *his7-1 *mutation is suppressed. Strain 1B-D1606 *sup45-107 *(*UPF1 sup45-107*) was used as a control of effective suppression of *his7-1 *mutation (as already reported [18]). *upf1Δ *strain did not grow on synthetic complete medium without histidine, demonstrating that there is no suppression of *his7-1 *in *upf1Δ *strain. Click here for file

Additional file 2 **Nonsense or missense *sup45 *mutations do not affect the steady-state levels of nonsense-containing *lys9-A21 *and *ade1-14 *mRNAs**. Northern blots were prepared with total RNA from wild-type strain 1B-D1606 (*SUP45*) and its *sup45 *mutant derivatives (bearing missense *sup45-103 *or nonsense *sup45-104 *mutations). Blots were hybridised with DNA probes that detected the *lys9-A21, ade1-14 *and *ACT1 *transcripts (*ACT1 *was used as a control). The fold increase in *lys9-A21/ACT1 *(upper panel) and *ade1-14/ACT1* (/lower panel) mRNA accumulation relative to such in wild-type strain are shown. The *lys9-A21/ACT1* and *ade1-14/ACT1* ratio in wild-type strain was set as 1.0. Click here for file

Additional file 3 **Deletion of *UPF1 *gene leads to increased viability of *sup45 *nonsense mutants**. Strains 3b-D1658 (*sup45Δ *pRS316/*SUP45*) and 3v-D1658 (*sup45Δ upf1Δ *pRS316/*SUP45*) all containing *SUP45 *deletion and pRS316/*SUP45-URA3 *plasmid were transformed with pRS315/*SUP45-LEU2 *plasmids carrying different *sup45 *mutant alleles. Following *sup45 *mutations were tested: 101, 102, 104, 105, 107 (nonsense) and 103 (missense). The growth of the transformants was tested by patching onto 5-FOA plates (**A**) or by plating 10^0^, and 10^-1 ^serial dilutions of overnight cultures (*left *to *right*) onto 5-FOA plates (**B**). The extent of cell growth on 5-FOA plates indicates the ability of the *sup45 *mutant alleles to support cell growth in the presence and absence of *UPF1 *gene. The same serially diluted cultures were also spotted on synthetic complete plates lacking leucine and uracil to estimate the total number of cells analyzed. (**C**) eRF1 and eRF3 protein levels in the clones selected on 5-FOA medium were analyzed by western blot. (*) indicates a non-specific band used as loading control. Click here for file
