# Supplementary figures and images for: Inactivation of NMD increases viability of sup45 nonsense mutants in Saccharomyces cerevisiae
Source: BMC Mol Biol. 2007 Aug 16;8:71. doi: 10.1186/1471-2199-8-71 (PMC2039749; doi:10.1186/1471-2199-8-71)

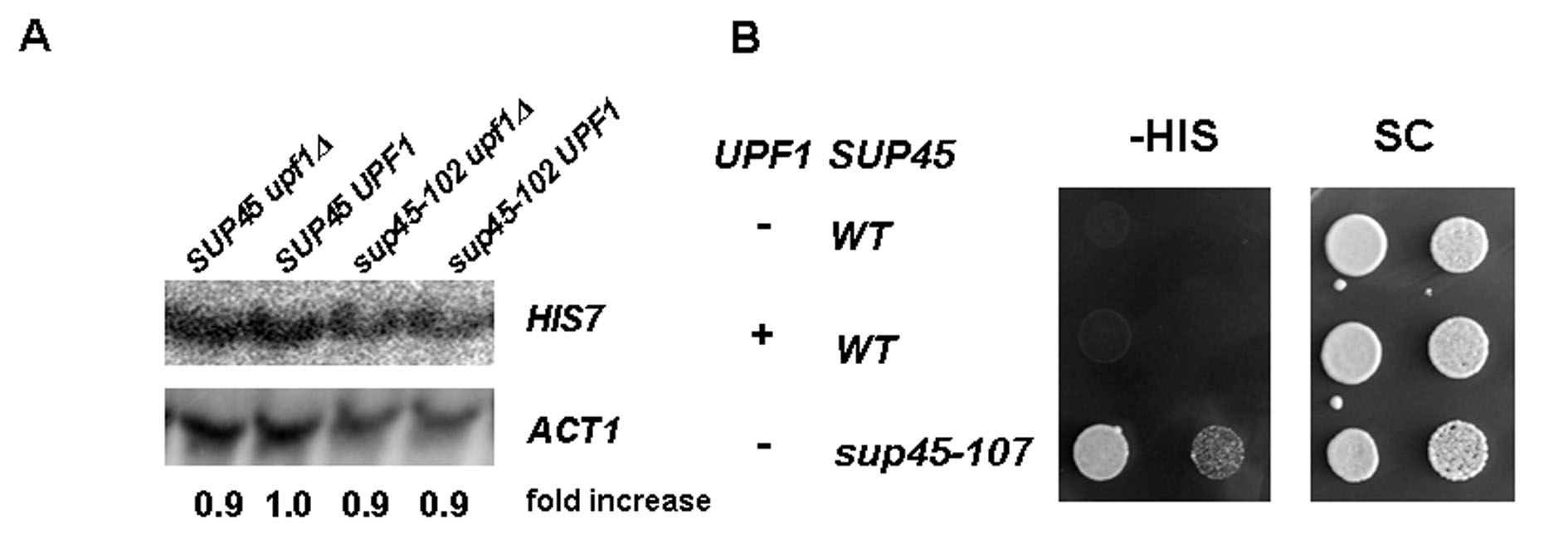

Supplement: Additional file 1 — A. Deletion of UPF1 and mutation of SUP45 do not affect the wild-type HIS7 mRNA level. Total RNA was isolated from strain 3v-D1658 (sup45Δ upf1Δ pRS315/SUP45) and its derivative (sup45Δ upf1Δ pRS315/sup45-102) transformed with pRS316 and pRS316/UPF1. Blots were hybridised with DNA probes that detected the wild-type HIS7 and ACT1 transcripts (ACT1 was used as a loading control). The fold increase in HIS7/ACT1 mRNA accumulation relative to such in wild-type strain is shown. The HIS7/ACT1 ratio in wild-type strain was set as 1.0. B. Deletion of UPF1 does not suppress his7-1 mutation. Strain 5B-D1645 (his7-1 upf1Δ) transformed with plasmids pRS316 and pRS316/UPF1, designated as (UPF1 -) and (UPF1 +), respectively. The growth of the transformants was tested by plating 100, and 101 serial dilutions of overnight cultures on a synthetic complete medium without histidine (-HIS). This strain bears his7-1 mutation and is unable to grow on such medium except if his7-1 mutation is suppressed. Strain 1B-D1606 sup45-107 (UPF1 sup45-107) was used as a control of effective suppression of his7-1 mutation (as already reported [18]). upf1Δ strain did not grow on synthetic complete medium without histidine, demonstrating that there is no suppression of his7-1 in upf1Δ strain. [file 1471-2199-8-71-S1.jpeg]

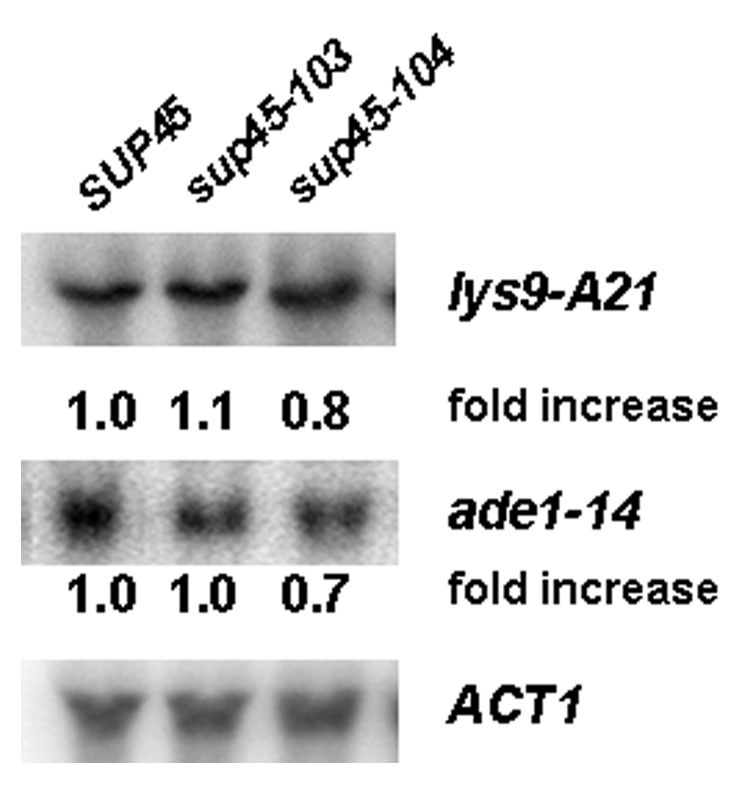

Supplement: Additional file 2 — Nonsense or missense sup45 mutations do not affect the steady-state levels of nonsense-containing lys9-A21 and ade1-14 mRNAs. Northern blots were prepared with total RNA from wild-type strain 1B-D1606 (SUP45) and its sup45 mutant derivatives (bearing missense sup45-103 or nonsense sup45-104 mutations). Blots were hybridised with DNA probes that detected the lys9-A21, ade1-14 and ACT1 transcripts (ACT1 was used as a control). The fold increase in lys9-A21/ACT1 (upper panel) and ade1-14/ACT1 (/lower panel) mRNA accumulation relative to such in wild-type strain are shown. The lys9-A21/ACT1 and ade1-14/ACT1 ratio in wild-type strain was set as 1.0. [file 1471-2199-8-71-S2.jpeg]

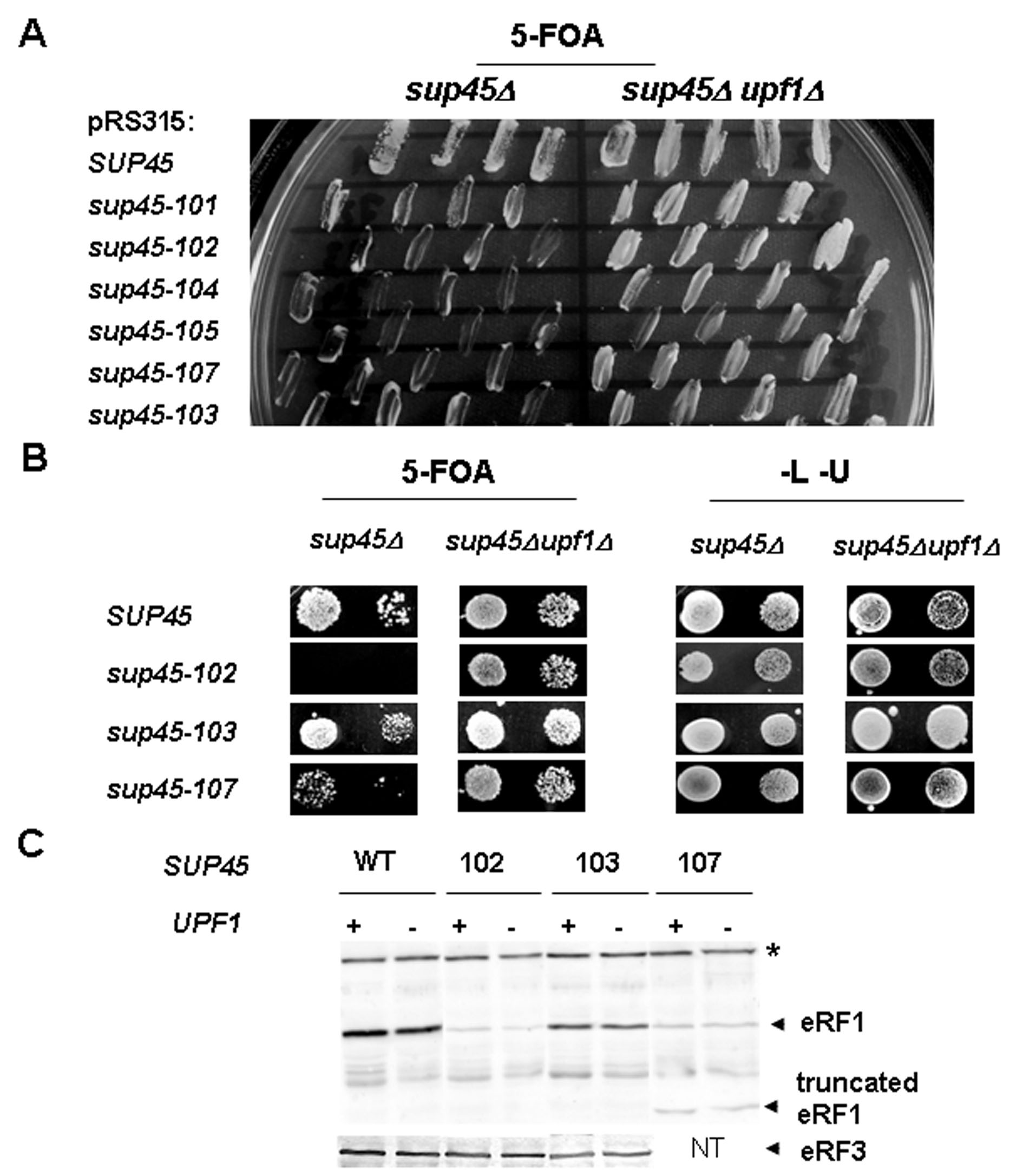

Supplement: Additional file 3 — Deletion of UPF1 gene leads to increased viability of sup45 nonsense mutants. Strains 3b-D1658 (sup45Δ pRS316/SUP45) and 3v-D1658 (sup45Δ upf1Δ pRS316/SUP45) all containing SUP45 deletion and pRS316/SUP45-URA3 plasmid were transformed with pRS315/SUP45-LEU2 plasmids carrying different sup45 mutant alleles. Following sup45 mutations were tested: 101, 102, 104, 105, 107 (nonsense) and 103 (missense). The growth of the transformants was tested by patching onto 5-FOA plates (A) or by plating 100, and 10-1 serial dilutions of overnight cultures (left to right) onto 5-FOA plates (B). The extent of cell growth on 5-FOA plates indicates the ability of the sup45 mutant alleles to support cell growth in the presence and absence of UPF1 gene. The same serially diluted cultures were also spotted on synthetic complete plates lacking leucine and uracil to estimate the total number of cells analyzed. (C) eRF1 and eRF3 protein levels in the clones selected on 5-FOA medium were analyzed by western blot. (*) indicates a non-specific band used as loading control. [file 1471-2199-8-71-S3.jpeg]
